# The Bacterial Microbiome of *Meloidogyne*-Based Disease Complex in Coffee and Tomato

**DOI:** 10.3389/fpls.2020.00136

**Published:** 2020-02-27

**Authors:** Araceli Lamelas, Damaris Desgarennes, Daniel López-Lima, Luc Villain, Alexandro Alonso-Sánchez, Alejandro Artacho, Amparo Latorre, Andrés Moya, Gloria Carrión

**Affiliations:** ^1^Red de Estudios Moleculares Avanzados and Red de Biodiversidad y Sistemática, Instituto de Ecología A. C., Xalapa, Mexico; ^2^CIRAD, UMR IPME, Montpellier, France; ^3^Joint Unit of Research in Genomics and Health, Foundation for the Promotion of Health and Biomedical Research in the Valencian Community (FISABIO) and Cavanilles Institute of Biodiversity and Evolutionary Biology, Universitat de València, Valencia, Spain; ^4^CIBER en Epidemiología y Salud Pública, Madrid, Spain; ^5^Institute for Integrative Systems Biology (I2SysBio), University of Valencia, Spanish National Research Council (CSIC-UVEG), Valencia, Spain

**Keywords:** corky root, pathobiome, *Meloidogyne enterolobii*, *Meloidogyne paranaensis*, functional profile

## Abstract

The *Meloidogyne-*based disease complexes (MDCs) are caused by the interaction of different root-knot nematode species and phytopathogenic fungi. These complexes are devastating several important crops worldwide including tomato and coffee. Despite their relevance, little is known about the role of the bacterial communities in the MDCs. In this study 16s rDNA gene sequencing was used to analyze the bacterial microbiome associated with healthy and infested roots, as well with females and eggs of *Meloidogyne enterolobii* and *M. paranaensis*, the causal agents of MDC in tomato and coffee, respectively. Each MDC pathosystems displayed a specific taxonomic diversity and relative abundances constituting a very complex system. The main bacterial drivers of the MDC infection process were identified for both crops at order level. While corky-root coffee samples presented an enrichment of Bacillales and Burkholderiales, the corcky-root tomato samples presented an enrichment on Saprospirales, Chthoniobacterales, Alteromonadales, and Xanthomonadales. At genus level, *Nocardia* was common to both systems, and it could be related to the development of tumor symptoms by altering both nematode and plant systems. Furthermore, we predicted the healthy metabolic profile of the roots microbiome and a shift that may result in an increment of activity of central metabolism and the presence of pathogenic genes in both crops.

## Introduction

Plants harbor a myriad of microorganisms out and inside their tissues, constituting specific microbiomes. Plant-associated microorganisms, mainly endophytes, play an important role in plant health and productivity, since they provide their hosts with essential functions (Kiers et al., 2003: Kiers and van der Heijden, 2006; Bulgarelli et al., 2012; Vandenkoornhuyse et al., 2015). Pathogens attack, results in the disturbance of the plant microbiome balance, possibly promoting diseases through cooperation or coinfection of different pathogens (Tollenaere et al., 2016; Vannier et al., 2019). The idea that disease could be caused by the combined action of more than one pathogen set the bases for the pathobiome concept, which describes the pathogen as a component of a complex microbial community (Ryan, 2013; Vayssier-Taussat et al., 2014; Lamichhane and Venturi, 2015; Jakuschkin et al., 2016; Brader et al., 2017).

The pathobiome theory considers the interaction between well adapted pathogens, known as keystone pathogens that modulate plant defenses and strongly affect plant microbiome composition; accessory pathogens, which find their niche in the pathobiome by providing nutritional or colonization support to keystone pathogens; and pathobionts, whose commensal or mutualistic relationship switches to pathogenic, in response to changes in the plant microbiome (Brader et al., 2017).

Considering this definition, the pathobiome concept contradicts the Koch and Hill's fundamental postulate “one microbe-one disease” (Ryan, 2013; Vayssier-Taussat et al., 2014), stating that disease is driven by a highly complex network in which microorganisms interact with each other influenced by diverse external factors (Lamichhane and Venturi, 2015; Brader et al., 2017).

Sedentary endoparasitic nematodes such as *Meloidogyne* spp. are very harmful plant parasites due to their high pathogenicity and their ability to overcome resistance in many hosts (Jones et al., 2013; Elling, 2013). Interactions of root-knot nematodes (RKN) with other phytopathogenic microorganisms can cause syndromes known as *Meloidogyne*-based disease complex (MDC), severely affecting world agricultural production (Wolfgang et al., 2019). MDC symptoms are characterized by the initial infection of RKN, causing the typical root swellings and knots. As the infection progresses, the concomitant presence of fungi and bacteria is observed along with nematodes in root tissues, which present more severe symptoms such as hyperplasia with deep and cracked cortical root tissues, having a cork-like appearance, leading to necrosis and atrophy of the root system (Koike et al., 2006; Taher et al., 2017; López-Lima et al., in press; Wolfgang et al., 2019). These symptoms, in the advanced stages of infection, are also commonly known as corky-root disease in coffee (Bertrand et al., 2000; Lopez-Lima et al., 2015; Villain et al., 2018). Consequently, water and nutrient flows are altered, causing significant damage to the plant including chlorosis, defoliation, necrosis of tip branches, and decreased production (Hajji-Hedfi et al., 2018).

The bacterial community is an important component of MDC, perhaps favoring disease development. Some groups of bacteria like *Herbaspirillum*, *Rickettsia*, *Chitinophaga*, and *Pedobacter* have been reported as symbionts of nematodes (Tian et al., 2011; Baquiran et al., 2013; Cheng et al., 2013). In contrast, other bacterial species such as *Pseudomonas* and *Commamonas* have been reported reducing nematode populations by inhibiting hatching or by nematicidal action (Siddiqui et al., 2006; Wolfgang et al., 2019). Root bacterial microbiome structure has been studied in some relevant crops affected by RKN and other pathogens with the aim of understanding their function in the agroecosystems and developing better management strategies (Busby et al., 2017; Finkel et al., 2017; Toju et al., 2018; Toju and Tanaka, 2019).

Particularly, severe effects of MDC have been reported in two crops of world importance: tomato (Sasanelli et al., 2008; Ganaie and Khan, 2011; Rizvi et al., 2015; Mwangi et al., 2019) and coffee (Alvarado, 1935; Carneiro et al., 1996; Carneiro et al., 2004; Villain et al., 2013; Lopez-Lima et al., 2015; Monteiro et al., 2016; Villain et al., 2018). In tomato MDC is caused by *M. incognita* and/or *M. enterolobii* interacting with *F. oxysporum*, *F. solani*, *Pyrenochaeta lycopersici*, and *Rhizoctonia solani* (Sasanelli et al., 2008; Ganaie and Khan, 2011; Rizvi et al., 2015; van Bruggen et al., 2016; Mwangi et al., 2019). In Mexico, *M. enterolobii* causes significant losses in due to its high virulence, which leads to plant death before completing the crop cycle. This is the case of cultivars carrying the resistant gene *Mi-1*, which confers resistance to *M. javanica*, *M. arenaria* and *M. incognita* (Kiewnick et al., 2008; Rosa et al., 2014; Martínez-Gallardo et al., 2015; Martínez-Gallardo et al., 2019).

In coffee, MDC is caused by the RKN *Meloidogyne paranaensis* or *M. arabicida* interacting with phytopathogenic fungi such as *Fusarium oxysporum* and *F. solani* (Bertrand et al., 2000; López-Lima et al., in press). In Mexico, Brazil and Guatemala, *M. paranaensis* is the RKN responsible for MDC, affecting both Arabica and Robusta crops (Carneiro et al., 1996; Carneiro et al., 2004; Villain et al., 2013; Lopez-Lima et al., 2015; Villain et al., 2018).

Notwithstanding, little is known about the bacteria associated with these MDCs and their role in the development of the disease in tomato and coffee crops. *M. enterolobii* and *M. paranaesis* are emerging species causing severe damage in tomato and coffee crops, respectively (Elling, 2013). These two pathosystems differ greatly, since tomato is a short-cycle crop mainly grown under greenhouse conditions and subjected to intensive management, while coffee is a woody perennial crop mainly grown within agroforestry systems.

In order to understand the pathogenicity process of a plant disease, the pathobiome components must be characterized. We are particularly interested in investigating the bacterial microbiome associated with the diseases caused by a root-knot nematode-fungi complex: *Meloidogyne*-based disease complex in tomato and coffee. The study aims were to: i) investigate the bacterial community of tomato and coffee, infested by MDC, and ii) determine whether bacterial community composition and function differ between healthy and infected tissues, as well as in different life stages involved in RKN and rhizospheric soil. This work will allow us to establish a fundamental understanding of the dynamics and ecological role of microbes in the pathobiome of *Meloidogyne*-based disease complex.

## Materials and Methods

### Study System and Sample Collection

Tomato (*Solanum lycopersicum)* samples were collected from a five months-old crop, cultivar Red Grape, located in Culiacan, Sinaloa, Mexico (24°55'59” N, 107°26'34” W at 67m.a.s.l). Coffee (*Coffea arabica*) samples were collected from a four years-old plantation, cultivar Costa Rica 95 in Jilotepec, Veracruz, Mexico (19°35'42” N, 93°53'01” W at 996 m.a.s.l). Plants were classified according to the progress of MDC symptoms and the root galling index (RGI, Coyne and Ross, 2014) from 1: no visible galling damage to 5: severe galling damage. 1) No symptoms: roots with no galling damage (RGI level 1, healthy root tissues), 2) Initial symptoms: roots with slight galling damage (RGI level 2), separated knots but without rot or necrosis (root nodule tissue), and 3) advanced symptoms: roots with heavy galling damage (RGI level 4–5), hyperplasia, continuous nodules, with deep and cracked cortical tissues and presence of rot and necrosis (corky-root tissues). Five plants were collected per plant species, from them healthy root tissue, root nodule tissue, corky-root tissue (in this study, corky-root refers to severely damaged root tissues in the last stage of the disease), female nematodes, egg masses, and rhizospheric soil were obtained (**Figure S1**, **Table S1**). Female nematodes and egg masses were obtained by dissecting corky roots. Nematodes were identified by species-specific SCAR markers (Randig et al., 2002; Tigano et al., 2010). *Meloidogyne enterolobii* and *M. paranaensis* were identified from tomato and coffee samples, respectively.

Females and eggs were washed with PBS (137 mM NaCl, 2.7 mM KCl, 10 mM Na_2_HPO_4_, pH 7.4) by slow pipetting. PBS females and eggs washing solution, containing external bacteria, was recovered (**Figure S1**, **Table S1**). All samples were stored at −20°C until processing.

### DNA Extraction, 16S rRNA Gene Amplification, and Sequencing

Female nematodes or eggs were ground using a sterile pestle in 1.5 mL tube after adding 1 mL of cold Extraction Buffer I (10 mM Tris, 60 mM NaCl, 5% Sucrose, 10 mM EDTA, pH = 7.8, sterilized and stored at 4°C). Then, 200 μL of Extraction Buffer II (300 mM Tris, 1.25% SDS, 5% Sucrose, 10 mM EDTA pH = 8.0) was added and mixed. Samples were then incubated for 30 min at 65°C. Afterward, 60 μL 3 M Potassium Acetate (pH = 4.8 to 5.2) were added, and the samples were incubated at −20 °C for 20 min, thawed at room temperature for 5 min and centrifuged at maximum speed. The recovered pellet was washed with 70% ethanol. Samples were air dried and resuspended in 35 μL of sterile molecular grade water. Root and soil DNA extraction was conducted using the Quick-DNA ™Fecal/Soil Microbe Miniprep kit (Zymo Research, Cat. No. D6010) following the manufacturer protocol.

To determine bacterial diversity, 16S ribosomal primers were designed from variable regions three and four and used for sequencing in an Illumina platform (Illumina, 2013) in the FISABIO foundation (Valencia, Spain). PCR reactions were conducted using total genomic DNA and Kappa polymerase (Kappa HiFi Hotstart Ready Mix, cat. No. KK2602, Kappa Biosystems). PCR products were cleaned up using magnetic beads (BeckmanCoulter Agencourt Ampure XP, cat. No. A63881) and resuspended in 10 mM Tris pH 8.5 or Resuspension buffer (RSB, Illumina Inc.).

Libraries were constructed using Nextera XT adapters (Illumina Inc.) using the same polymerase and cleaned again using magnetic beads.

Libraries were quantified by fluorescence using a Qubit DNA HS kit, library quality was analyzed using a Tapestation 2200 or Bioanalyzer 2100 and pooled to an equimolar ratio to be sequenced in a 2X300 bp MiSeq Run. The raw data was deposited in NCBI´s sra archive under bioproject accession number PRJNA591631.

### Bioinformatic and Statistical Analysis

All the reads with a quality below 20 were filtrated by Prinseq-lite program (Schmieder and Edwards, 2011). R1 and R2 fastq files were joined by fastq-join from the ea-tools suit. Sequences were then checked for chimeras with Mothur (Schloss et al., 2009) using Greengenes 13-8 database (http://greengenes.lbl.gov/). The reads were clustered at 97% in OTUs (Operational Taxonomic Units) using uclust algorithm as implemented in the script pick_open_reference_otus.py from QIIME 1.9.1 (Caporaso et al., 2010; Lan et al., 2012). The taxonomy assignment was done by RDP method (Lan et al., 2012) against Greengenes 13-8 database. Each sample was further manually filtered to remove OTUs missing in at least four out of the five samples in the same treatment, with a frequency lower than 0.01% and with homology to the chloroplast, archaea, and mitochondrial sequences (Supporting Information **Table S2**). Alpha diversity estimations were calculated using the next scripts: multiple_rarefactions.py, alpha_diversity.py, collate_alpha.py and make_rarefaction_plots.py from QIIME 1.9.1. Rarefaction curves were generated by subsampling the OTU table with step increments of 10 and the sampling depth of 1,500 reads. The alpha diversity indexes Chao1, Shannon, and Simpson and the number of OTUs by Treatment, were plotted by “*vegan*” and “*stats*” for R v.3.3.1 (Oksanen et al., 2016; R Core Team, 2016). We applied the Pairwise Wilcox test tested to determine significant differences between the diversity indexes of the treatments.

The relative taxonomic abundance of the Samples and the Treatment was represented by collapsed histogram plotted by “*RColorBrewer*” and “*ggplot2*” libraries for R v.3.3.1 (Wickham, 2009; Neuwirth, 2014). The data table contained the relative frequency of the most frequent OTUs and the collapse frequency of the others by its closest common taxa. To visualize the relative abundance of the most frequent OTUs a heat map per treatment was constructed by “*ape*”, “*gplots*”, “*vegan*” and “*RColorBrewer*” libraries for R v.3.3.1 (Paradis et al., 2004; Warnes et al., 2016). The OTUs with a frequencies higher than 0.1% were represented and the soil samples were not considered.

To represent dissimilarities across the samples, we performed a nonmetric multidimensional scaling (NMDS) using the Bray-Curtis distances matrix. In the NMDS plot, the distance between objects represents the relative dissimilarity between them. The measure of the goodness of fit of the final NMDS plot was represented by the *stress* value, which indicates the match between inter-object distance and dissimilarity. The closer the *stress* value is to zero, the better the ordination (Manly, 1986; Quinn and Keough, 2002). Finally, the effect of crop and sample type was evaluated with a permutational multivariate ANOVA (PERMANOVA) using the Bray-Curtis dissimilarities (Anderson, 2001; Anderson and Walsh, 2013), the significance threshold for PERMANOVA was set at *p* < 0.001. All the analyses were performed using “*vegan*” and “*mass*” for R v.3.3.1 (Oksanen et al., 2016; Venables and Ripley, 2002).

We predict the metagenome functional profiling of the different samples base on the Kyoto Encyclopaedia of Genes and Genomes (KEGG) pathway by PICRUSt. To assess the accuracy of the PICRUSt predictions, the Nearest Sequenced Taxon Index was calculated for each sample and treatment (Langille et al., 2013; **Tables S3** and **S4**). The effect of crop and sample type on KEGG orthology (KOs) was evaluated with a PERMANOVA using a Bray-Curtis dissimilarities matrix, which was previously calculated considering the relative abundance of functional categories in all samples. The significance threshold for PERMANOVA was set at *p* ≤ 0.001. When PERMANOVA was significant, differences between samples were determined with multiple pairwise comparisons using a Wilcoxon test with FDR correction set at *p* ≤ 0.05. All the analyses were performed using “*vegan*” and “*mass*” for R v.3.3.1.

We generated two cluster dendrograms based on the Bray-Curtis distance matrix estimating of the OTUs taxonomic abundance filtered at 0.01%, the KOs abundance predicted by PICRUSt by “*vegan”* for R v.3.3.1.

We made a graphical representation of the main drivers of the healthy and Corky/knot conditions using relative abundance data at order level.

Based on the functional dendrogram results we defined three main clusters, and we built Venn diagrams of the specific or shared KOs between corky clusters and the healthy cluster, and among coffee and tomato corky clusters and the soil cluster.

## Results

### Diversity and Species Richness of Bacterial Community

A total of 2,131,578 reads were sequenced by Illumina PE platform. The quality check removed 20% of the reads obtaining an average of 53,846 quality read filters per sample (max = 2,002,726, min = 1,426) (**Table S1**). The sequencing depth quality was confirmed by estimating the Chao, Shannon, and Simpson alpha diversity indexes and rarefaction curves of the number of observed OTUs, where all the curves reflected a saturated sampling (**Figure S2**).

The OTUs richness and diversity were calculated using Simpson and Shannon indexes. In general, in both crops the number of observed OTUs differed significantly among sample types (ANOVA, Tomato: *F*_(7,__40)_ = 8.72, *p* = 5.77e-06; Coffee: *F*_(7,__40)_ = 25.97, *p* = 1.73e-11), with higher values in soil samples than in root samples, and higher values in root samples than in nematode samples (Supporting Information **Figure S3**). Moreover, the coffee samples exhibited significantly higher values than tomato samples, with a maximum value of 310.45 ± 19.45 (observed OTUs) for coffee soil samples and a minimum value of 81.8 ± 51.9 (observed OTUs) for female wash solution from tomato samples (*F*_(7,__40)_ = 4.43, *p* = 0.0394). Regarding the diversity indexes, significant differences were only detected across coffee samples (Shannon: *F*_(7,__40)_ = 12.58, *p* = 1.25e-07, Simpson: *F*_(7,__40)_ = 2.83, *p* = 0.206), registering the maximum diversity values in soil samples (7.147 ± 0.277) and the minimum in female wash solution samples (4.252 ± 0.609) (Supporting Information **Figure S3**).

**Figure 1:**
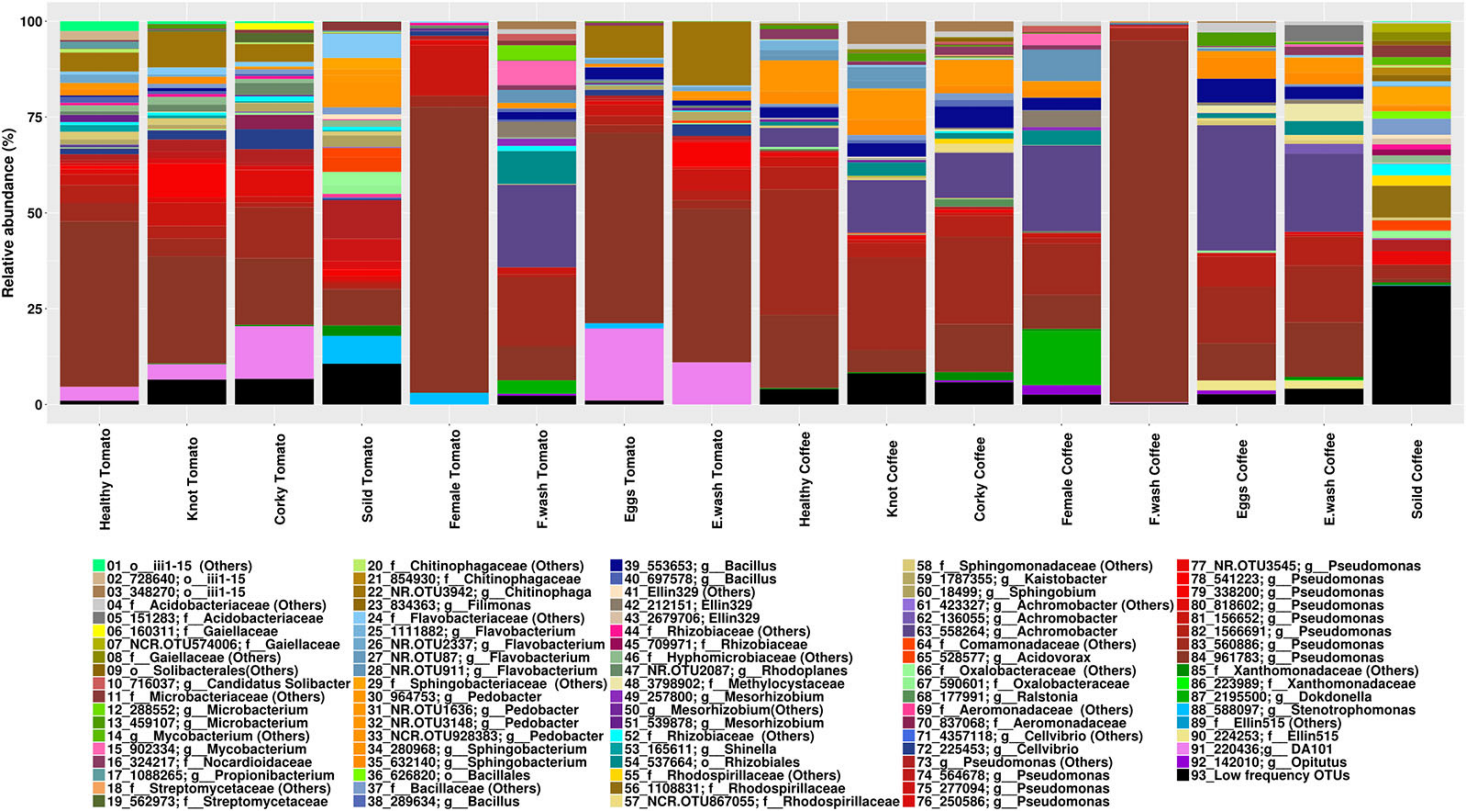
Relative taxonomic abundance by treatment represented by the most frequent OTUs, the least frequent taxa are collapsed in others OTUs.

**Figure 2:**
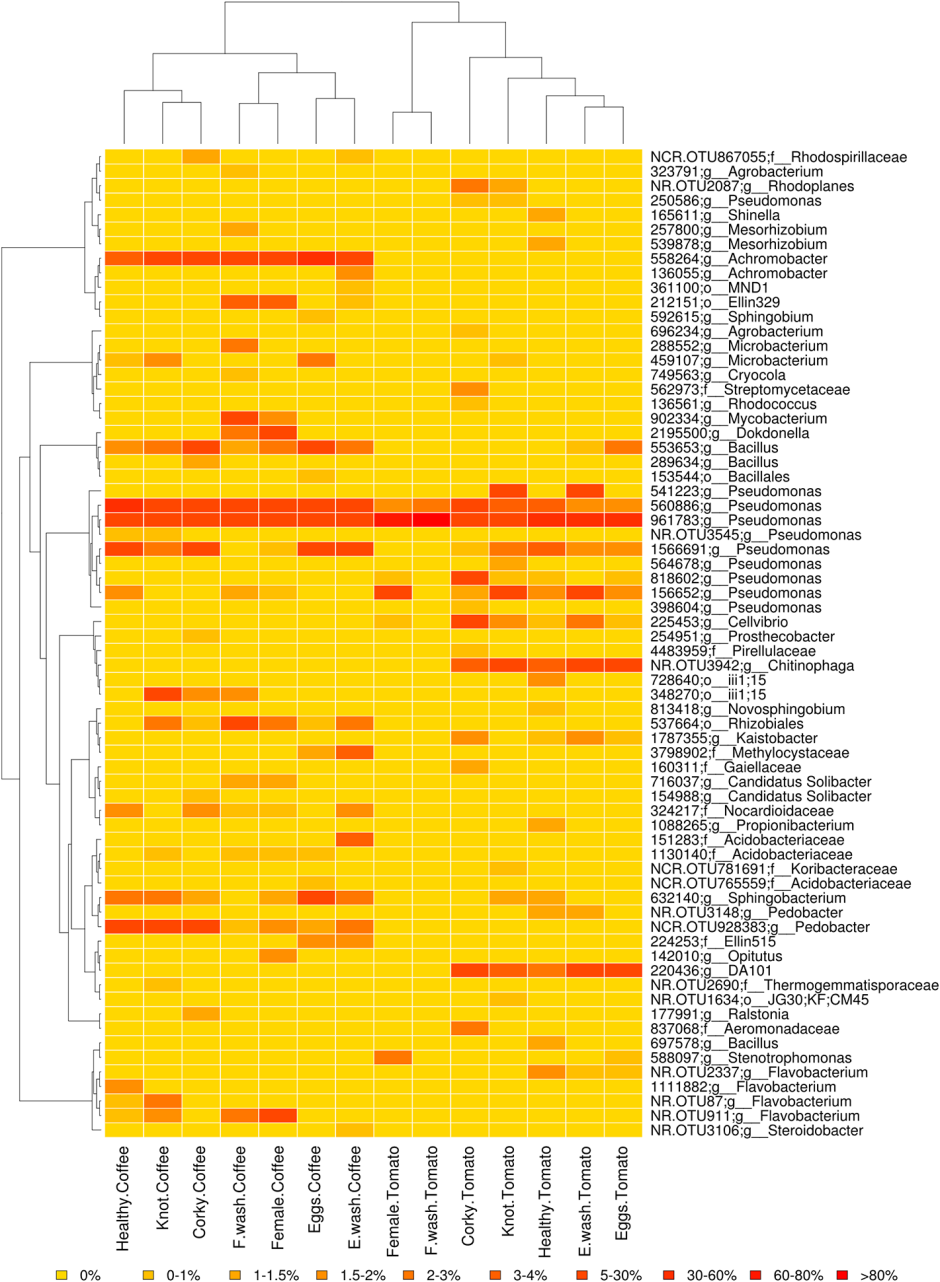
Relative abundance heat map of the OTUs with a frequency higher than 0.1% by treatment. The soil samples are not taken into account. The dendrogram on the left shows the phylogenetic relationship between the OTUs, the dendrogram on the top illustrate the relationship between the samples based on Ray distance. Yellow, low relative abundance; orange, high relative abundance.

The high-quality reads were clustered over 881 OTUs, most of them belonging to the phylum Proteobacteria (49%), Bacteroidetes (14%), and Actinobacteria (10%). The relative abundance of major genera and families by treatment is shown in **Figure 1** (Supporting Information **Figure S4**). The genera *Pseudomonas* and *Flavobacterium* had the highest relative abundance with a total of 8.07% and 5.67%, respectively. The genera shared for all samples were *Pseudomonas* and *Bacillus*, while at OTUs level 560886 and 961783 (*Pseudomonas*) were the only ones common to all samples. The core microbiome of the tomato samples consisted of the genera *Cellvibrio* and *Rhodoplanes*, while *Microbacterium*, *Achromobacter*, *Pedobacter*, and *Flavobacterium* were the genera present in core microbiome of coffee. The OTUs 558264 (*Achromobacter*) and NCR.OTU928383 (*Pedobacter*) were shared by all the coffee samples (**Figure 2**). No common core microbiome was found among nematode samples. Root samples however, shared the genera *Novosphingobium*, *Sphingobacterium*, and *Janthinobacterium*. In the case of *Janthinobacterium*, we found 10 OTUs with a relative frequency lower than 0.1%. Specifically, the corky root had the genera *Nocardia* in common, although in low frequencies, 0.001% in corky root tomato and 0.009% in corky root coffee. Soil and Corky tomato samples shared 22 OTUs belonged to the genera *Pseudomonas, Sphingobacterium, Flavobacterium, Rhodoplanes, Cellvibrio, Shinella, Luteolibacter, Brevundimonas, Janthinobacterium, Chitinophaga, Bacillus*, and *Agrobacterium*, while Soil and Corky coffee samples shared 10 OTUs belonged to the genera *Pedobacter, Janthinobacterium*, and *Candidatus Solibacter*.

**Figure 3:**
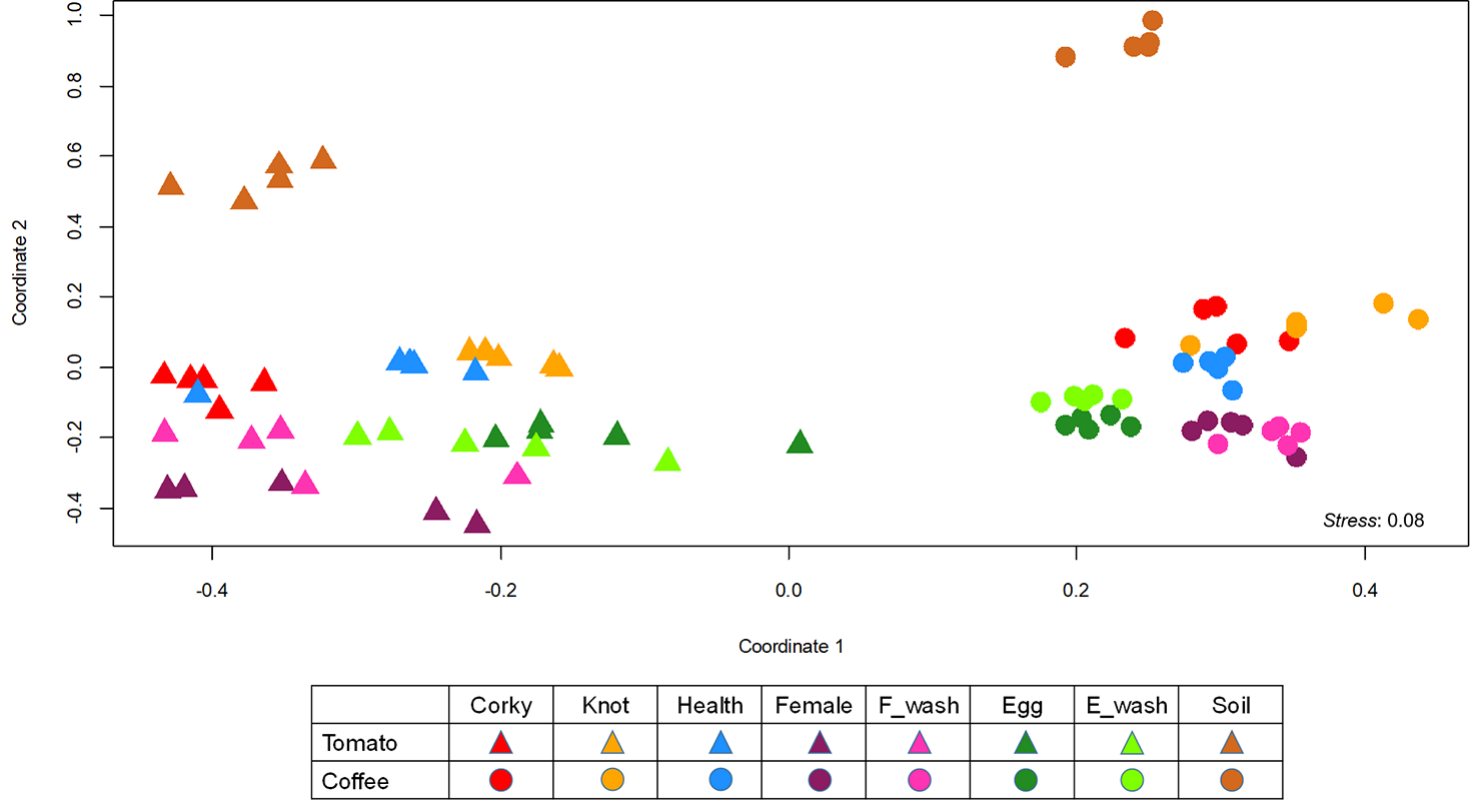
Non-metric multidimensional scaling (NMDS) analysis of all the samples. NMDS plot for Bray–Curtis dissimilarities of the bacterial communities associated with tomato and coffee.

Permutational ANOVA (PERMANOVA) and NMDS analysis (NMDS) of the bacterial community revealed that both factors, crop species and sample type, clearly determine the bacterial community assemblage (**Figure 3**, **Table 1**). The microbiome structure of all coffee samples differed significantly from the all the tomato samples (PERMANOVA, *F_(1,__80)_* = 213.987, *R^2^* = 0.27180, *p* = 0.001; **Figure 3**). Moreover, the soil samples associated to each crop, differed from the other samples (Tomato: *F_(1,__40)_* = 27.584, *R^2^* = 0.85783, *p* = 0.001; Coffee: *F_(1,__40)_* = 25.753, *R*^2^ = 0.84925, *p* = 0.001; **Figure 3**). A split analysis of all tomato or coffee samples showed significant differences between root and nematode samples (**Figure S5**) and among the different disease developmental stages (knots vs. corky tissues) and the nematode stages (eggs vs. females) (**Figure S5**).

**Table 1:** Permutational multivariate ANOVA of the bacterial communities associated with different sample types of coffee and tomato.

Factor^a^	*F*	*R*^2^	*P*
*Global*			
Crop_1,__64_	206,690	0.27095	0.001
Treatment_7,__64_	41,087	0.37703	0.001
Crop:treatment_7,__64_	29,218	0.26812	0.001
*Tomato*			
Treatment_7,__32_	26,627	0.85347	0.001
*Coffee*			
Treatment_7,__32_	26,277	0.85181	0.001

a Subscript numbers indicate the degrees of freedom and residuals of each F test.

The microbiome structure was modified by nematode infection, leading to the depletion of Pseudomonadales (Gammaproteobacteria) and Sphingobacteriales (Sphingobacteria) and the enrichment of Actinomycetales (Actinobacteria), in both crops. The microbiome structure variation of both crops differed in the taxonomic orders. The corky-root coffee samples exhibited an enrichment of Bacillales (Bacilli) and Burkholderiales (Betaproteobacteria) in comparison with healthy samples, while in corky-root tomato samples Saprospirales (Saprospirae), Chthoniobacterales (Spartobacteria), Alteromonadales (Gammaproteobacteria), and Xanthomonadales (Gammaproteobacteria) were enriched in comparison with healthy samples and Rhodobacterales were slightly depleted in corky-root samples. The pairwise comparisons of relative abundance between Healthy, Knot, and Corky coffee samples within the order Rhodobacterales were significant with a p-value of 0.03 (**Figure S6**).

### Predictive Functional Analysis of the Bacterial Microbiome

**Figure 4:**
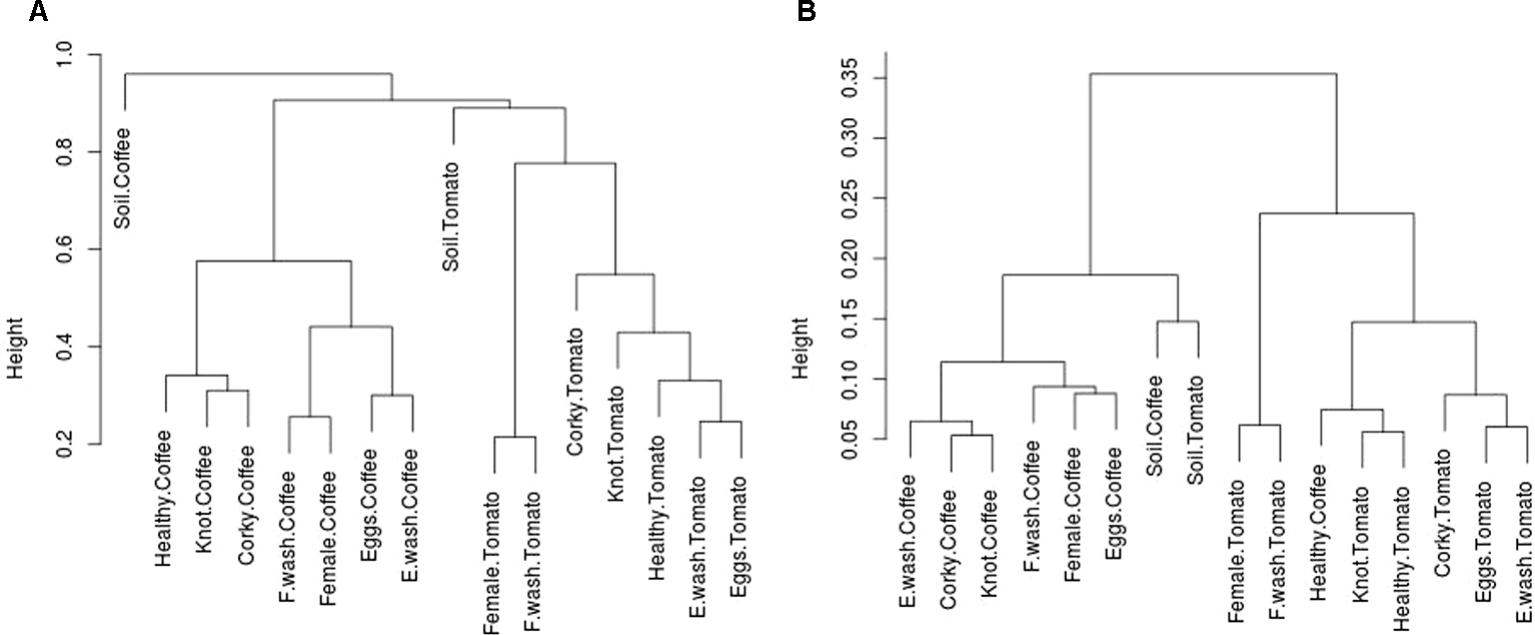
Cluster dendrogram of dissimilarity matrix of **(A)** OTUs relative abundance and **(B)** KO's abundance predicted by PICRUSt.

The association of the microbial community with a metabolic profile was assessed by PICRUST analysis. We predicted the gene content of the bacterial microbiome and construct a dendrogram based on taxonomic and metabolic dissimilarities (**Figure 4**, **Tables 1** and **2**). The results showed that, while at a taxonomic level the samples grouped primarily based on the crop species (tomato vs. coffee plant) and within each of both groups, based on the origin of the sample (nematode stage vs. root source); at a functional level (KO), in both cases (tomato and coffee plants), the PBS wash solution eggs and the corky root samples grouped together, with the particularity that within the coffee cluster, knot samples were also present in this cluster and that in the tomato samples, egg samples belonged to this cluster. The healthy root samples of both crops clustered together along with knot tomato samples. Regarding coffee and tomato soil samples, at a taxonomic level they both clearly separated and clustered with their relative root and nematode crop samples, whereas at a functional level (KO), they clustered together and with the coffee infested roots and nematode stages samples.

**Table 2:** Permutational multivariate ANOVA of the metabolic functionality Kyoto Encyclopaedia of Genes and Genomes categories of the bacterial communities associated with different sample types of coffee and tomato crops.

Factor^a^	*F*	*R^2^*	*P*
*Global*			
Crop_1,__64_	2.7409	0.17921	0.001
Treatment_7,__64_	5.4902	0.25139	0.001
Crop:treatment_7,__64_	3.291	0.15069	0.001
*Tomato*			
Treatment_7,__32_	4.9528	0.52002	0.001
*Coffee*			
Treatment_7,__32_	2.9209	0.38985	0.001

a Subscript numbers indicate the degrees of freedom and residuals of each F test.

**Figure 5:**
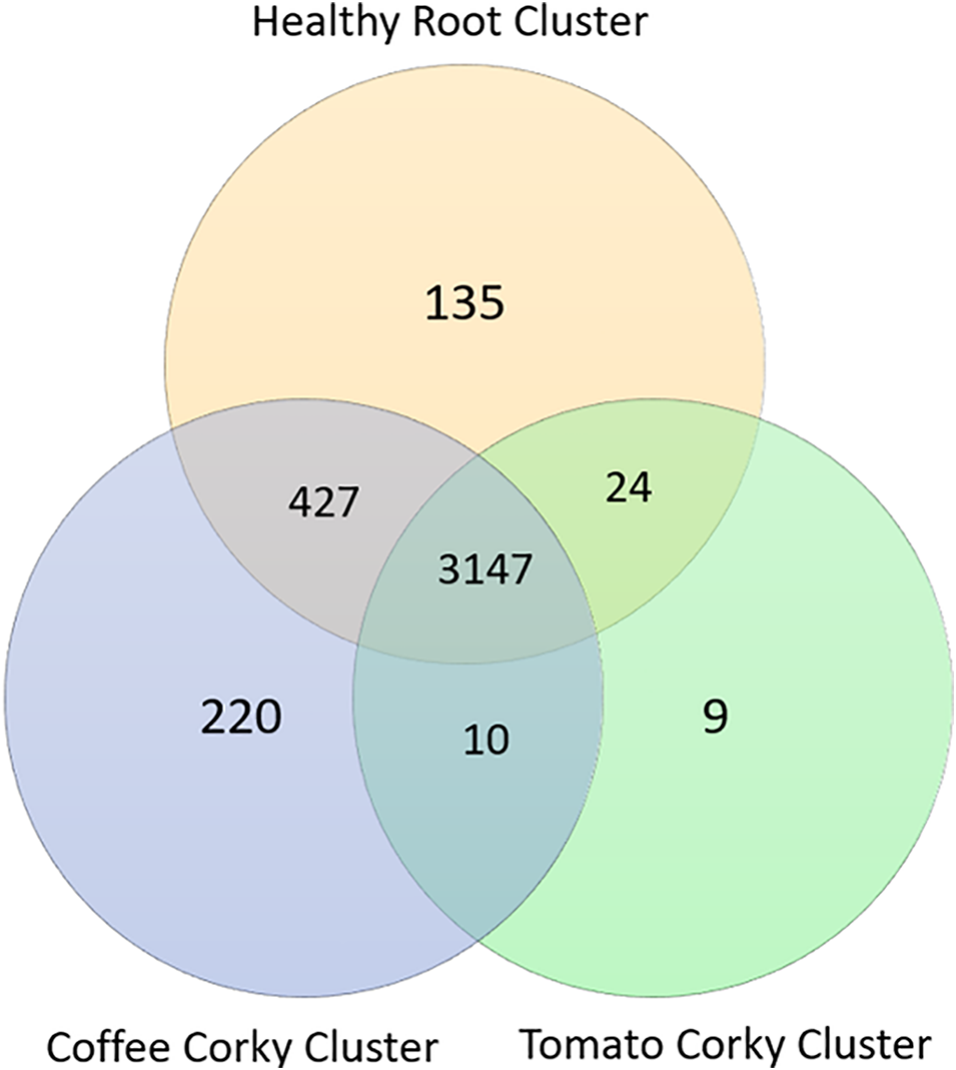
Venn diagram of the KO numbers specific to ot shared by the coffee and tomato corky root clusters and the healthy root cluster.

The Healthy Root Cluster (healthy tomato roots, healthy coffee roots, and tomato root-knot) had 6,071 KOs, the Coffee Cork Cluster (corky roots, root-knots, wash solution egg) had 6,211 KOs and the Tomato Cork Cluster (corky roots, eggs, wash solution egg) had 5,264 KOs shared 3,147 KOs and had 135, 220, and 9 specific KOs, respectively. The metabolic biomarkers that defined the healthy root stage were defined by the presence of 135 KOs, which were classified principally in the following functional categories: Membrane transport (42 KOs), Carbohydrate metabolism (13 KOs), Cellular processes (eight KOs), Glycan biosynthesis and metabolism (eight KOs), Metabolism of cofactors and vitamins (seven KOs), and Poorly characterized functions (26 KOs) (**Figure 5**).

**Figure 6:**
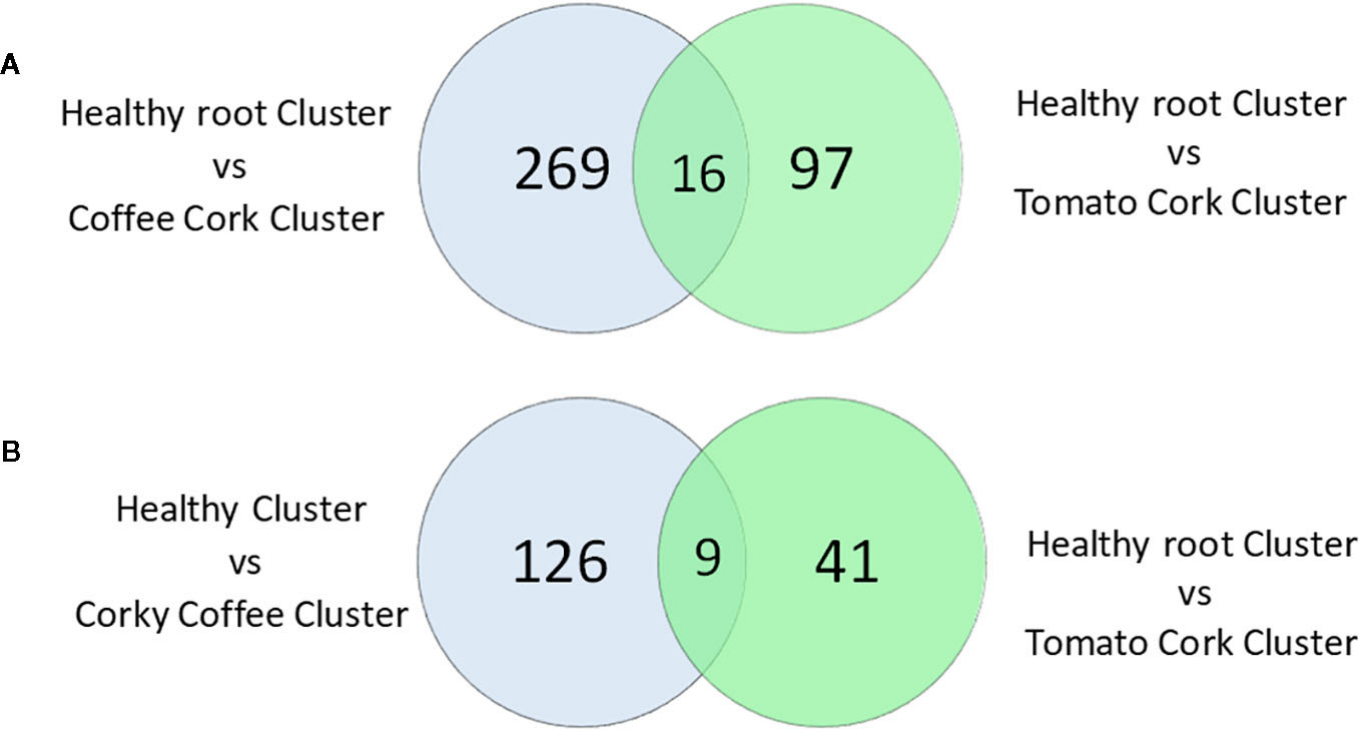
Venn diagram of the KO numbers **(A)** with higher significant relative frequency, and **(B)** with lower significant relative frequency, on the healthy root cluster relative to coffee corky root and tomato corky root clusters.

Moreover, the samples of the Healthy Cluster had 285 KOs with higher relative frequency and 135 KOs with a lower significant relative abundance compared to the Corky Coffee Cluster, while the Healthy Cluster samples had 113 KOs with significantly higher relative abundance and 50 KOs with a significantly lower relative abundance compared to the samples of the Corky Tomato Cluster (**Table S5**, **Figure 6**). The 285 mentioned KOs with higher relative frequency in the healthy cluster vs. in the corky coffee cluster classified principally in the following functional categories: Membrane transport (94 KOs), Amino acid metabolism (36 KOs), Transcription (36 KOs), Glycan biosynthesis and metabolism (26 KOs), Carbohydrate metabolism (25 KOs), Cellular processes and signaling (11 KOs), and Unclassified (61 KOs). The other 135 with lower relative frequency classified in almost the same functional categories: Membrane transport (105 KOs), Carbohydrate metabolism (61 KOs), Amino acid metabolism (50 KOs), Transcription (45 KOs), Cellular processes and signaling (13 KOs), and Unclassified (83 KOs). Only the metabolic functions Replication and repair and Glycan biosynthesis and metabolism showed higher numbers in the Healthy Cluster samples (15 and 26 KOs, respectively) than in the Corky Coffee Cluster samples (9 and 14 KOs, respectively). Regarding the 113 KOs with higher frequency in Healthy Cluster compared to Corky Tomato Cluster, they classified mainly in the functional categories: Membrane transport (20 KOs), Carbohydrate metabolism (20 KOs), Amino acid metabolism (19 KOs), Xenobiotics biodegradation and metabolism (19 KOs), Cellular processes and signaling (four KOs), and Unclassified (19 KOs), while the other 50 KOs with lower frequency classified on Metabolism of cofactors and vitamins (six KOs), Amino acid metabolism (five KOs) Cellular processes and signaling (six KOs), and Unclassified (six KOs). While the metabolic profile of the Corky Coffee Cluster samples was defined by the increase in the KOs number of the different metabolic categories compared to the Healthy Cluster samples, the Corky Tomato Cluster samples were defined by a decrease of the same functional categories.

Corky stage has been characterized by the presence of 10 specific KOs, which were classified principally in the following functional categories: Cellular processes and signaling (KOs 4), Membrane transport (3), Unclassified (3). Compared to Corky Coffee stages, only 220 specific KOs were found and classified in: Membrane transport (34 KOs), Carbohydrate metabolism (23 KOs), Amino acid metabolism (15 KOs), Cellular processes and signaling (14 KOs), Enzyme families (13 KOs), Xenobiotics biodegradation and metabolism (seven KOs), and Unclassified (57 KOs). In Corky Tomato stages, only nine specific KOs were found, involved principally in Membrane transport (three KOs), Cellular processes (signaling, 2) and Unclassified (four KOs).

A specific analysis of the metabolic profile shared by the Soil Clusters and both Cork Cluster, showed that Cork coffee Cluster and Soil Cluster shared 290 KOs, with 14.14% (67 KOs) involved in Environmental Information Processing and 60.34% (286 KOs) involved in Metabolism. The Cluster Cork Tomato and Soil Cluster shared 128KO's which were involved in Environmental Information Processing 35,96% (64 KOs) and 35,96% (64 KOs) involved on Metabolism (**Figure S7**).

## Discussion

In this study, we used 16S rDNA gene Illumina sequencing to analyze the microbial communities associated with *M. enterolobii* and *M. paranaensis* and different disease stages of the MDC in tomato and coffee field plants. The microbiome isolated from soil, healthy roots, infested roots, and different nematode developmental stages of each crop showed a very complex and distinct structure.

The two crop systems (tomato vs. coffee) differed in their bacterial community, alpha diversity and OTUs richness, all these variables were higher in coffee than in tomato samples. Similarly, the crop type clearly affected the composition of MDC bacterial microbiome, as revealed by NMDS and PERMANOVA. Coffee samples came from perennial plantations, grown under agroforestry systems, holding several tree shade species and with minor use of fertilizers and pesticides (Gordon et al., 2007; González-Zamora et al., 2016). Moreover, tillage is not employed, leading to the establishment of many soil microbiota species (Legrand et al., 2018; Dube et al., 2019). By contrast, tomato, an annual crop with short cycle, is known by its intensive cropping systems, with high demand of agrochemicals (both fertilizers and pesticides), applied before and during the growing cycle (Pérez-Olvera et al., 2011; Pratt and Ortega, 2019). Previous studies have shown that plant management or status has a clear effect on the associated microbiome (Coleman-Derr et al., 2016; Hartman et al., 2018; Compant et al., 2019) and that intensively grown crops have a lower diversity of associated plant and soil microbiota than agroecosystems subjected to less aggressive agricultural practices (Rathore et al., 2019; Saleem et al., 2019).

We consider that the significant differences of microbiota diversity found between the two studied crop systems could be related with the management practices, leading to the establishment of a more diverse bacterial community in the coffee agroecosystem.

Moreover, we found significantly higher alpha diversity and OTUs richness in root samples than in nematode samples. However, for both crops, there was no significant differences between disease stages or nematode life cycle stages. These results differ with those observed by Wolfgang et al. (2019), who describe decreasing alpha diversity during the root disease process. Conversely, we observed a non-significant increasing diversity in disease stages in both crops.

We identified Pseudomonadales, Sphingobacteriales, and Actinomycetales as the main common drivers of the MDC implicated in the infection process for both crops. Additionally, we observed crop-specific drivers of the MDC infection process: Bacillales and Burkholderiales in coffee samples and Saprospirales, Chthoniobacterales, Alteromonadales, and Xanthomonadales in tomato samples. Few similarities with the results published by Tian et al. (2015) and Wolfgang et al. (2019) were observed. Our results and the two cited studies only agree in the richness reduction of Pseudomonadales in the corky-root samples in comparison with the healthy ones. The order Pseudomonadales contains species listed as opportunistic parasites (Castillo et al., 2017); however, other species in the same order have shown nematicide effects by the production of compounds such as cyanide (Siddiqui et al., 2006). The discrepancies regarding the main drivers observed in these studies may be due to cultivar differences (Rybakova et al., 2017). These differences could be explained by the diverse sets of available microorganisms with varied abilities to attach to the nematode cuticle. We also consider that the rhizobiome composition could be affected by the physicochemical conditions of the soil (Bonito et al., 2019; Saad et al., 2019). Wolfgang et al. (2019) proposed that changes in physiological conditions of plant cells is the cause by a microbial structure shift, and this is influenced by the ability of some bacteria to adhere to the nematode cuticle (Elhady et al., 2017). In addition, it has been shown that the rhizosphere microbiome may influence the severity of RKN infection by promoting pathogens in infested soils and suppressing pathogens in uninfected soils (Zhou et al., 2019).

At the genera level, only *Nocardia* was shared by the corky-root samples. Species of this genus are found worldwide in soils rich in organic matter, some of which are plant pathogenic (Xing et al., 2011; Bai et al., 2016; Conville et al., 2017). *Nocardia* sp. is the causal agent of the disease known as tobacco false broomrape, which causes cracked tumors in the main and secondary roots, resembling the hyperplasia produced in the advanced stages of the MDC in tomato and coffee (Morán et al., 2013; Morán-Gómez et al., 2018). Moreover, *Agrobacterium* was externally recovered from *M. enterolobii* female and tomato corky-roots. Species of this genus have virulence genes that affect the response of the plant cells to growth regulators (auxins and cytokinins) and induce uncontrolled cell division, which results in tissue proliferation and gall formation (Bourras et al., 2015; Lacroix and Citovsky, 2019). Thus, the genera *Nocardia* and *Agrobacterium*, associated with RKN microbiome reported in this and other studies (Cao et al., 2015), may contribute to the formation of corky root symptoms in the MDC.

Additionally, *M. enterolobii* produces the MeTCTP (translationally controlled tumor protein) effector that promotes parasitism by suppressing programmed cell death in host plants (Zhuo et al., 2017). The combined effect of the tumor caused by the nematode and the bacteria could explain the remarkably wide range of hosts and severity of symptoms observed in plants infested with this nematode (Elling, 2013; Jones et al., 2013); however, this requires further investigation.

The grouping pattern based on the microbiome metabolic prediction differs from the grouping pattern based on the taxonomic information. At taxonomic level we observed two big clusters based on the crop of origin (Tomato vs. Coffee) and the type of sample (Roots vs. Nematodes), leaving the soil samples alone as an external group. By contrast, we observed three metabolic clusters: the Healthy Cluster (Healthy coffee and tomato roots plus Tomato Root-Knot samples); the Coffee Corky cluster (Coffee Corky-Roots and Root-Knots plus Coffee PBS washout nematode Eggs samples); and the Tomato Corky cluster (Tomato Corky-Roots plus Tomato nematode Eggs and PBS washout Eggs samples).

Metabolic functions of the microbiota associated with the soil samples of both crops were comparable, as well as the metabolic profile of healthy roots of both crops. The metabolic functional categories enriched in Corky samples versus soil samples, considering the KOs analysis, were associated to pathogenicity, and include cell signaling and regulation and membrane transport, and other categories like carbohydrate metabolism, amino acid metabolism, cofactor and vitamins metabolism, family enzymes, and transcription factors related with the increase in the metabolic activity in the nematode feeding site. Our metagenomics analyses conducted in the two phathosystems (Tomato- *M. enterolobii*, and Coffee- *M. paranaensis*) suggest that root infection by the nematode is, on the contrary, not linked to a specific microbiome community, but is associated with a metabolic profile shift characterized by an increase in pathogenicity and central metabolism functional categories, which is consistent with Tian et al. (2015) observations.

Additionally, one of the defense mechanisms of the coffee plant toward the attack of *Meloidogyne* spp. is the accumulation of phenolic compounds at the feeding site to limit their reproduction (Albuquerque et al., 2010; Silva et al., 2010). Our results, indicate that corky-roots of coffee plants had more metabolic profiles that encode enzymes involved in the pathway of benzoate degradation (aminobenzoate and benzoate) than healthy coffee roots. Benzoate is an intermediate in the pathway of anaerobic metabolism of aromatic compounds, i.e. phenolic compounds (Carmona et al., 2009). It is possible that some of the bacteria associated with coffee corky-root prevent the accumulation of phenolic compounds at the feeding site of *M. paranaensis* and contribute to the infection process, as occurs in other pathosystems (Cheng et al., 2013).

Our results indicate that, during nematode pathogenesis and nematode feeding site induction in plants, the MDC infection process could be mediated by particular metabolic microbial categories at a functional level, even though the two MDCs studied here involved different RKN species and unspecific associated bacterial taxa. Nonetheless, further studies are required to gain a deeper understanding of the bacterial functional roles in the MDC infection process.

## Data Availability Statement

The datasets generated for this study can be found in NCBI using accession number PRJNA591631.

## Author Contributions

GC, DL-L, ArL designed the experiments. DL-L and LV collected and prepared the samples. DL-L, ArL extracted the DNA. AM and AmL performed the sequencing. ArL and AA performed the bioinformatic analysis. DD performed the statistical analyses. GC, ArL, DD, DL-L, LV, and AA-S wrote the manuscript. GC, ArL, DD, DL-L, LV, AA-S, AM, AmL read, revised, and approved the manuscript.

## Funding

The research leading to these results has received funding from the Research Projects of High Strategic Value for Society (Project 2003530920) founded by Institute of Ecology A. C (INECOL).

## Conflict of Interest

The authors declare that the research was conducted in the absence of any commercial or financial relationships that could be construed as a potential conflict of interest.
