# Supplementary material for: The Bacterial Microbiome of Meloidogyne-Based Disease Complex in Coffee and Tomato
Source: Front Plant Sci. 2020 Feb 27;11:136. doi: 10.3389/fpls.2020.00136 (PMC7056832; doi:10.3389/fpls.2020.00136)
Supplement: Supplementary file 1 [file DataSheet_1.docx]

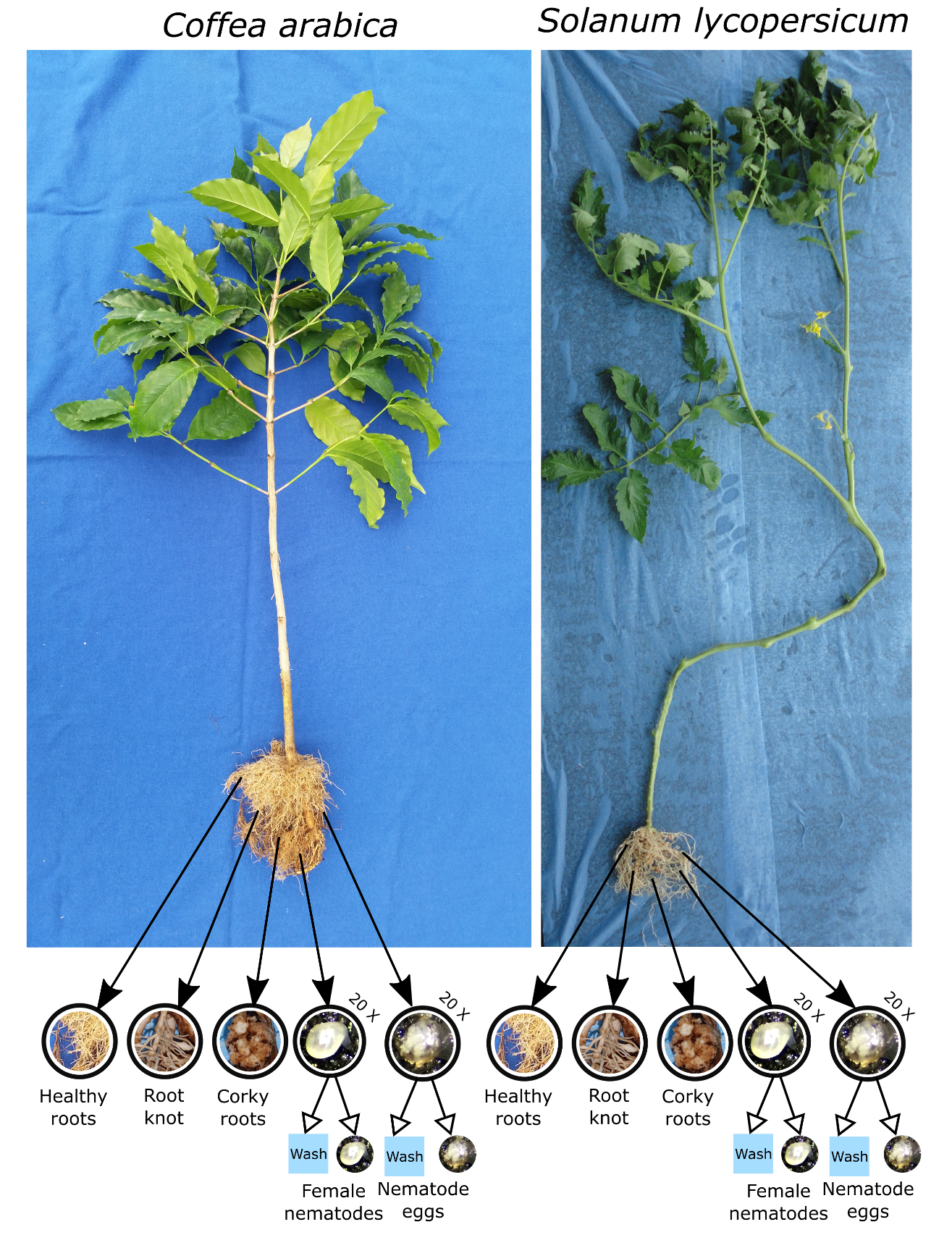


**Fig. S1 Procurement sampling scheme**. Procurement sampling scheme. Samples from healthy root tissue, root knots, and corky root tissue, as well as 20 adult female nematodes and 20 egg sacks were collected from Coffee (*Coffea arabica*) and Tomato (*Solanum lycopersicum*) plants. Female nematodes and egg sacks were washed with a PBS solution. The wash solution from eggs, females and root samples were collected for DNA extraction.

**
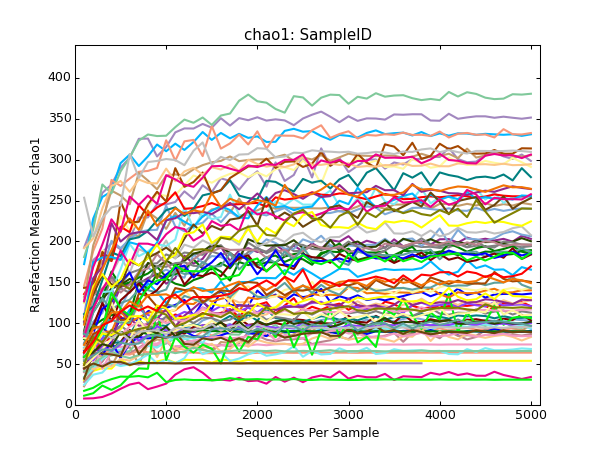

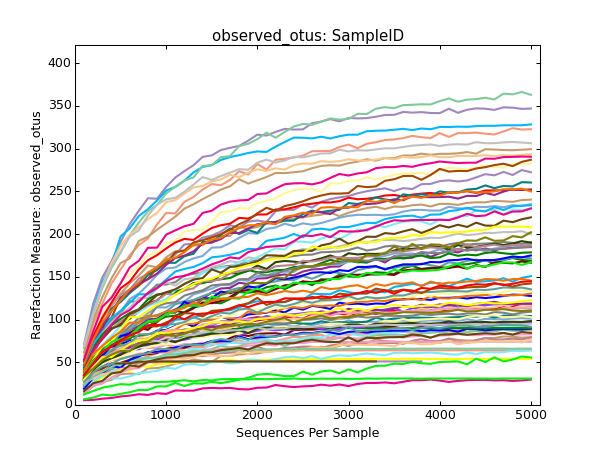

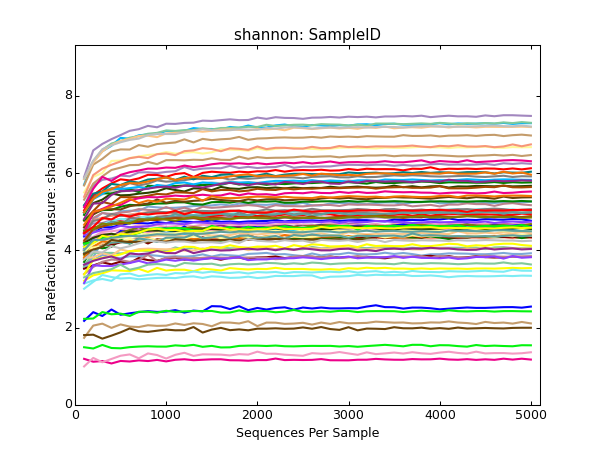

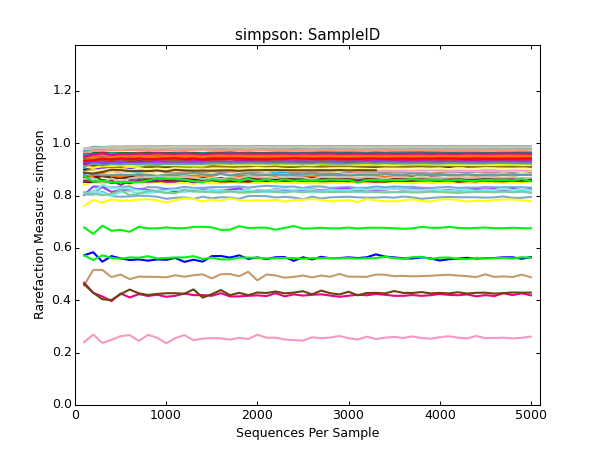
**

**b)**

**d)**

**a)**

**c)**

**Fig. S2** Rarefaction curves of bacterial OTUS, after filtering, sequenced in these study samples.

**
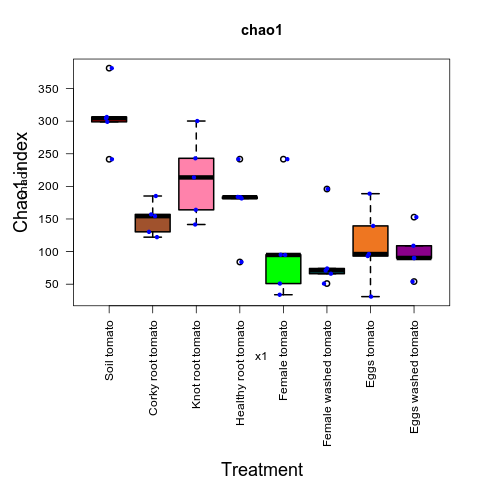

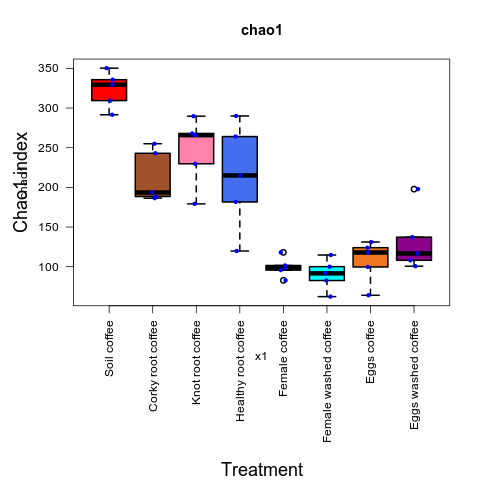
**

ab

a

**b)**

**a)**

a

b

b

b

Number of Observed OTUs

bc

bc

bc

Number of Observed OTUs

bc

bc

c

c

c

c

c

(ANOVA: *F_(7, 40)_*= 29.11, *p*= 3.75e-12)

(ANOVA: *F_(7, 40)_*= 9.16, *p*= 3.55e-06)

**
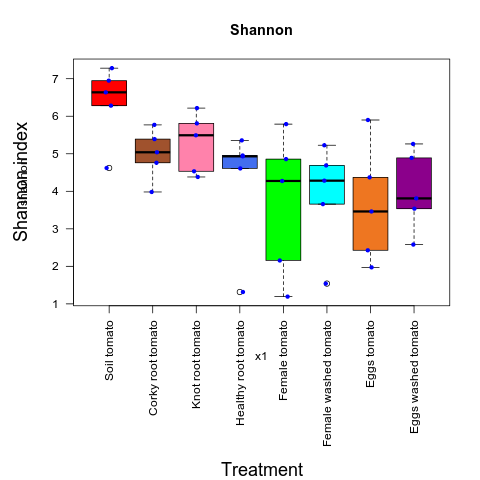

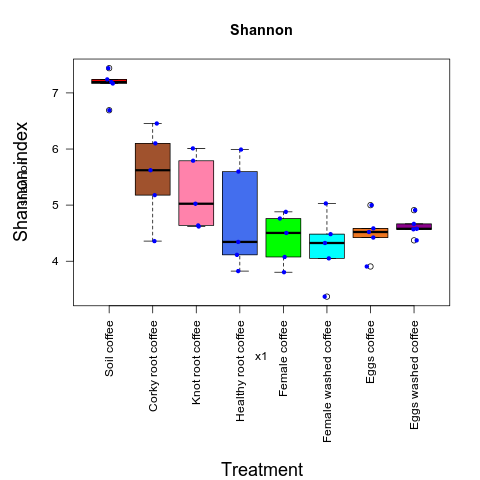
**

**d)**

**c)**

bc

bc

b

a

Shannon index

c

bc

Shannon index

bc

bc

(ANOVA: *F_(7, 40)_*= 2.59, *p*= 0.31)

(ANOVA: *F_(7, 40)_*= 12.58, *p*= 1.25e-07)

**
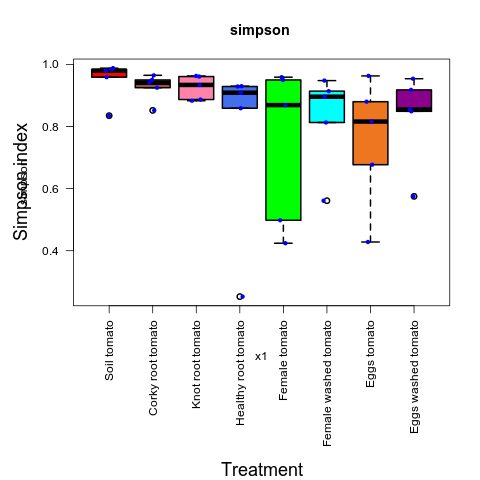

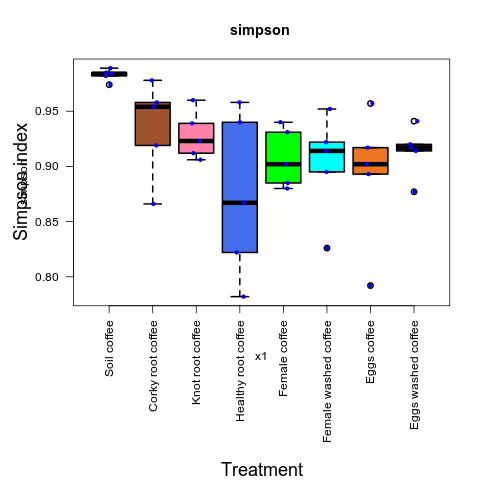
**

ab

**e)**

**f)**

ab

ab

ab

ab

b

a

b

Simpson index

Simpson index

(ANOVA: *F_(7, 40)_*= 2.83, *p*= 0.0206)

(ANOVA: *F_(7, 40)_*= 1.11, *p*= 0.383)

**Fig. S3** Box plot of: Number of Observed OTUs: a) of coffee samples, b) of tomato samples; Shannon index: c) of coffee samples, d) of tomato samples; Simpson index e) of coffee samples, f) tomato samples, per treatment. Distinct letters mean significant differences between samples after an ANOVA and a Tukey test. Graphics without letters mean no significant differences between samples.


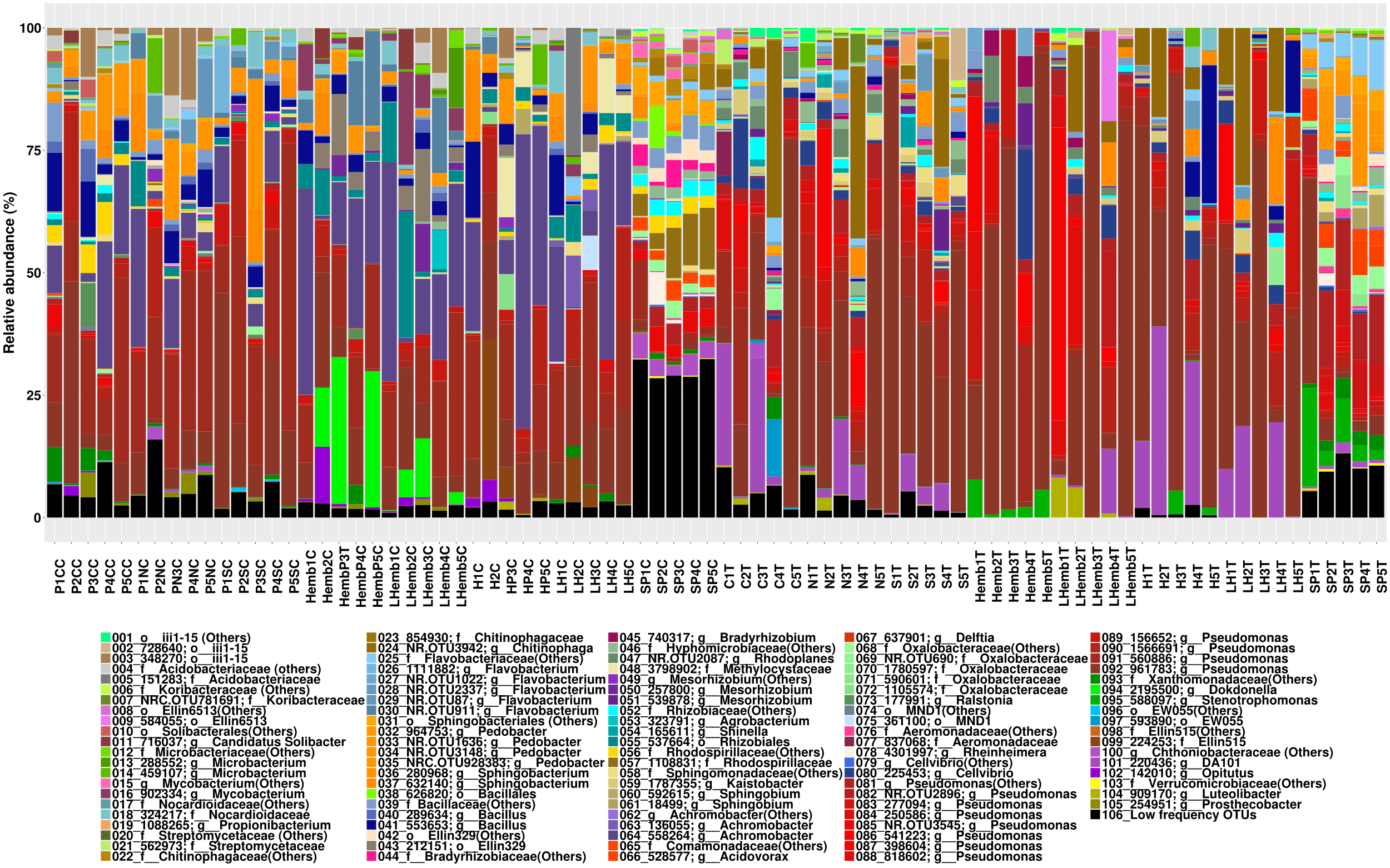


**Fig. S4** Relative taxonomic abundance by samples representing the most frequent OTUs and the least frequent taxa are collapsed in others OTUs.

a)

b)

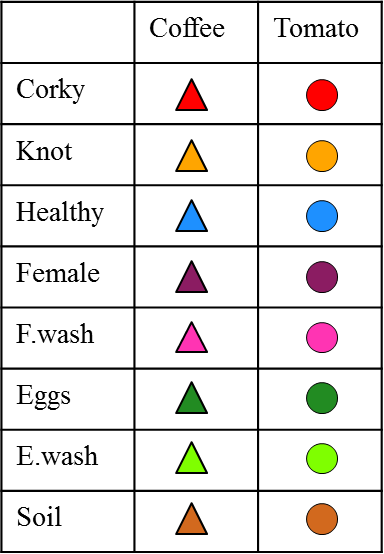


Tomato

Coffee

c)

**Fig. S5 NMDS analysis of both crops.** Plots display all samples of a) tomato, b) coffee and c) coffee without soil samples.


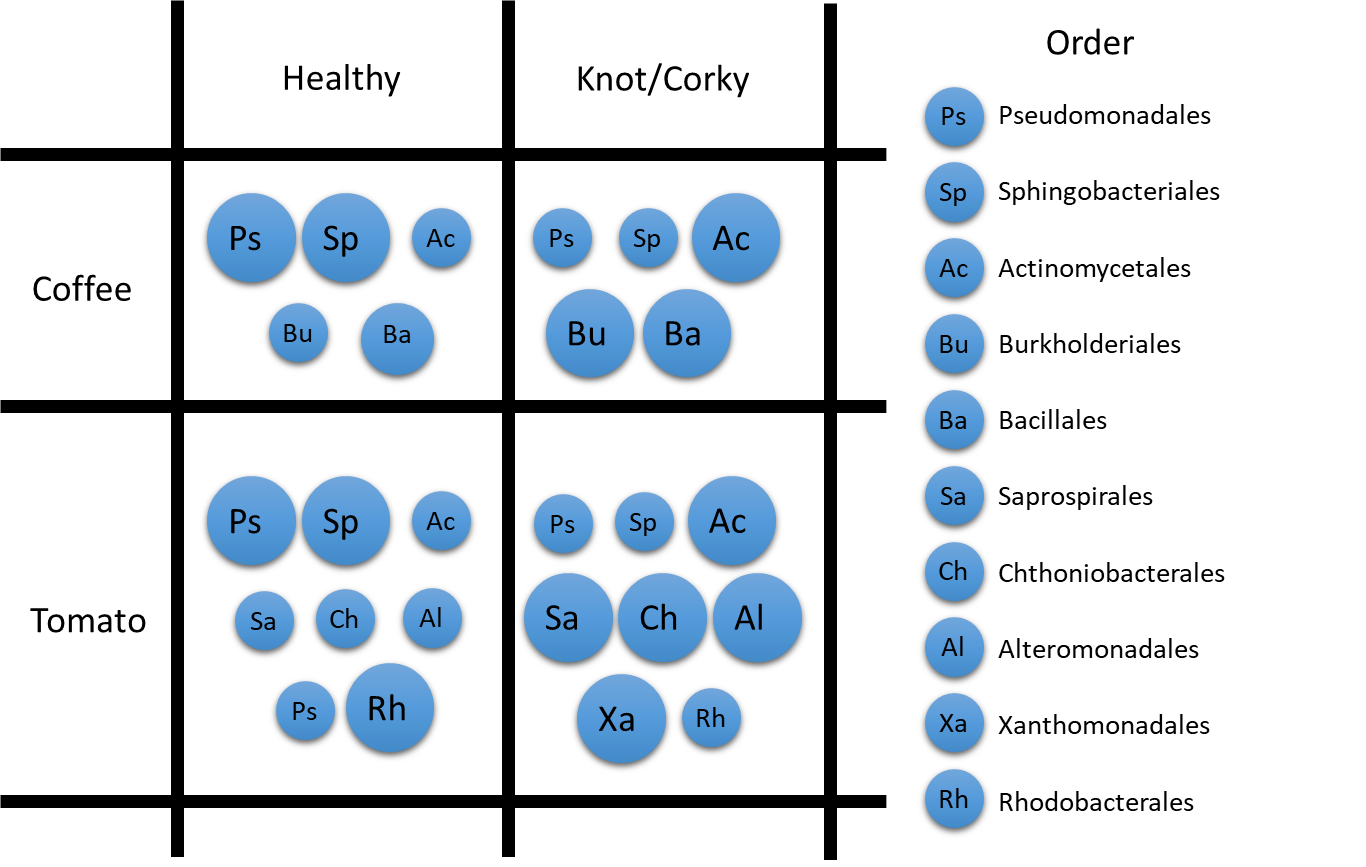


**Fig. S6 Principal main drivers.** Representation of the principal orders which relative abundance differ between the healthy and Corky/knot samples.

1092

11

128

11

Tomato Cork Cluster

Coffee Cork Cluster

Soil Cluster

**Corky Coffee Cluster *vs.* Soil cluster**

**Corky Tomato Cluster *vs.* Soil cluster**

**Fig. S7** Venn diagram of the KO numbers specific to or shared by the coffee and tomato corky root clusters and the soil cluster.
